# Preferred Barefoot Step Frequency is Influenced by Factors Beyond Minimizing Metabolic Rate

**DOI:** 10.1038/srep23243

**Published:** 2016-03-18

**Authors:** Matthew B. Yandell, Karl E. Zelik

**Affiliations:** 1 Department of Mechanical Engineering, Vanderbilt University, Nashville, TN 37212, USA; 2 Department of Biomedical Engineering, Department of Physical Medicine & Rehabilitation, Vanderbilt University, Nashville, TN 37212, USA

## Abstract

Humans tend to increase their step frequency in barefoot walking, as compared to shod walking at the same speed. Based on prior studies and the energy minimization hypothesis we predicted that people make this adjustment to minimize metabolic cost. We performed an experiment quantifying barefoot walking metabolic rate at different step frequencies, specifically comparing preferred barefoot to preferred shod step frequency. We found that subjects increased their preferred frequency when walking barefoot at 1.4 m/s (~123 vs. ~117 steps/min shod, *P* = 2e-5). However, average barefoot walking metabolic rates at the preferred barefoot and shod step frequencies were not significantly different (*P* = 0.40). Instead, we observed subject-specific trends: five subjects consistently reduced (−8% average), and three subjects consistently increased (+10% average) their metabolic rate at preferred barefoot vs. preferred shod frequency. Thus, it does not appear that people ubiquitously select a barefoot step frequency that minimizes metabolic rate. We concluded that preferred barefoot step frequency is influenced by factors beyond minimizing metabolic rate, such as shoe properties and/or perceived comfort. Our results highlight the subject-specific nature of locomotor adaptations and how averaging data across subjects may obscure meaningful trends. Alternative experimental designs may be needed to better understand individual adaptations.

Humans value economy of locomotion, and prior studies suggest that people tend to adopt a step frequency that minimizes metabolic rate at a given walking speed^1^,^2^,^3^,^4^,^5^,^6^. However, this energy minimization hypothesis related to step frequency is based on the study of shod walking. During barefoot walking, subjects increase their self-selected step frequency (as compared to shod walking at the same speed^7^,^8^,^9^), but the reason for this increase has not been explained.

One potential reason for the increase in barefoot step frequency (relative to shod) is that removing the shoes changes the gait optimization (e.g., because the mass, length or other properties of the shoe no longer have an effect), shifting the frequency at which minimum metabolic energy is expended for a given speed. For instance, simple walking models have demonstrated that the human speed-step frequency relationship can be reasonably approximated as a cost trade-off between performing mechanical work during stance phase and the exerting effort to swing the leg^10^. Thus, the removal of shoe mass during barefoot walking could potentially reduce the leg swing cost, resulting in a new metabolically-optimal step frequency. Previous studies have demonstrated that people can quickly adapt their step frequency to find a new metabolic minimum when locomotor demands are altered^11^,^12^. An alternate possibility is that humans adopt a faster step frequency when walking barefoot even though it does not minimize metabolic rate. The locomotor pattern during barefoot gait may result from a more complicated cost function, involving more than metabolic rate^6^,^13^,^14^,^15^,^16^. For instance, a person might prefer to walk at a less economical step frequency while barefoot in order to avoid the discomfort of large heel impacts. People appear to be willing to trade economy for comfort^17^, and previous evidence suggests that people change their motor behavior when running barefoot^18^,^19^,^20^ and when running or landing onto surfaces with varying levels of cushioning or compliance^21^,^22^,^23^.

The purpose of this study was to test the first possibility, that people increase their step frequency while barefoot in order to minimize the metabolic cost of walking. We performed an experiment to quantify the metabolic rate of walking barefoot at different step frequencies, and specifically to compare the metabolic rate when walking barefoot at an individual’s preferred barefoot step frequency vs. their preferred shod step frequency.

## Methods

We sought to compare the rate of metabolic energy expenditure for individuals walking barefoot at their barefoot vs. shod self-selected (SS) step frequency. We tested 10 subjects (21.5 ± 3.2 years old, 69.6 ± 13.5 kg, 171.8 ± 9.7 cm height, mean ±s.d.) during level walking on an instrumented treadmill. This sample size resolves mean differences >2.5% for this paired t-test experimental design, assuming power = 0.9, alpha = 0.05 and 2% standard deviation for within day metabolic measurements^24^. This study was approved by the Vanderbilt University Institutional Review Board and all subjects gave informed written consent prior to participation. Experiments were carried out in accordance with approved guidelines.

### Pre-Test

Each subject performed pre-test treadmill acclimation/training trials at sequentially increasing speeds from 0.6 to 1.4 m/s. These trials were first performed shod, using the subject’s own tennis shoes. Once subjects reached a steady gait cycle at each speed, we measured their step frequency, and defined this as their shod SS step frequency. This acclimation protocol was then repeated for barefoot walking, and each subject’s barefoot SS frequency was calculated. Two of the 10 subjects only completed acclimation trials at the experimental testing speed of 1.4 m/s, while the remaining 8 participants completed acclimation at all speeds.

### Metabolic Testing

Subjects performed 10 walking trials while matching the step frequency of a metronome and one trial standing at rest. Previous work found that using a metronome to enforce a desired step frequency did not significantly impact metabolic results, compared to walking at the same step frequency without a metronome^5^. For all trials, a metabolic system (Cosmed K4b2, Rome, Italy) was used to measure the subject’s oxygen uptake and carbon dioxide production rates for a minimum of 5.5 minutes. These steady-state estimates are relevant to both short and long duration bouts of walking. Although the length of “real-world” walking periods is typically much less than 5.5 minutes^25^, it has been estimated that the majority of the energy is still consumed at or near steady-speed walking during these bouts^26^. In addition, ground reaction force data were also recorded at 1000 Hz via the split-belt treadmill (Bertec, Columbus, OH, USA) and used to aid interpretation of results. All studies began in the morning, and subjects were instructed to refrain from eating breakfast prior to the start of the study. At the start of the study subjects completed acclimation trials and their SS step frequencies were measured while both barefoot and shod, as detailed above. Metabolic data were then recorded while the subject was at rest (Trial 0, Table 1). Following this, subjects performed AB conditions, in which “A” represents barefoot walking at the barefoot SS frequency, and “B” represents barefoot walking at shod SS frequency (Trials 1–2, Table 1). We also sought to more carefully characterize the systematic changes in metabolic cost due to increasing or decreasing barefoot walking step frequency. Therefore, after the AB conditions subjects performed 5 additional randomized conditions, matching step frequencies from −15% to +10% relative to their barefoot SS frequency (−15%, −10%, −5% [~shod SS step frequency], +5%, +10%). We selected this range to include trials approximately ±10% from both the shod and barefoot SS step frequencies. As a point of reference, one shod trial, walking at the shod SS frequency, was also randomized in the order and tested (Trials 3–8, Table 1). The subjects then repeated AB conditions at the end of the study (Trials 9–10, Table 1), but with the order reversed (BA). Some participants completed the trials in AB-BA order, and the remainder completed the trials in BA-AB order to help eliminate order bias. We had participants complete these identical conditions at the start and end of the study to compare barefoot walking at their barefoot vs. shod SS frequencies (Table 1). We limited the total number of trials to 10 to avoid subject fatigue.

### Post Test

After completion of the metabolic trials, each subject’s (except 1) SS barefoot and shod step frequencies were measured again (without the metronome) at the nominal study speed of 1.4 m/s to confirm consistency (Table 1).

### Data Analysis

Results were analyzed to determine if the metabolic rate during barefoot walking was reduced at the barefoot SS frequency as compared to the shod SS frequency. Gross metabolic rate was computed from the metabolic data corresponding to 50–90% of the trial duration (2.74 ± 0.38 min., mean ± s.d.), and used to calculate metabolic power based on the equation given by Au^27^, derived from Brockway^28^. Net metabolic power was then calculated by subtracting out the power during standing rest and normalized by subject mass. Statistical comparisons were performed using an analysis of variance with Holm-Sidak step-down correction and significance level of α = 0.05 for the parameter sweeps, and a two-tailed paired t-test using significance level of α = 0.05 for all other comparisons. Individual limb center-of-mass (COM) work rate was computed as the dot product of filtered (25 Hz, 3rd order, low-pass, zero lag) 3-dimensional ground reaction forces with the COM velocity^29^,^30^ and used as a supplementary measure of gait biomechanics. Mechanical cost of transport was calculated to estimate positive COM work per meter. Prior studies suggest that under certain circumstances increases in mechanical cost of transport tend to correlate with increases in metabolic cost^31^,^32^. To investigate this potential relationship a linear regression (with 95% confidence interval) was fit to these data, and the correlation coefficient was computed.

## Results

**Figure 1:**
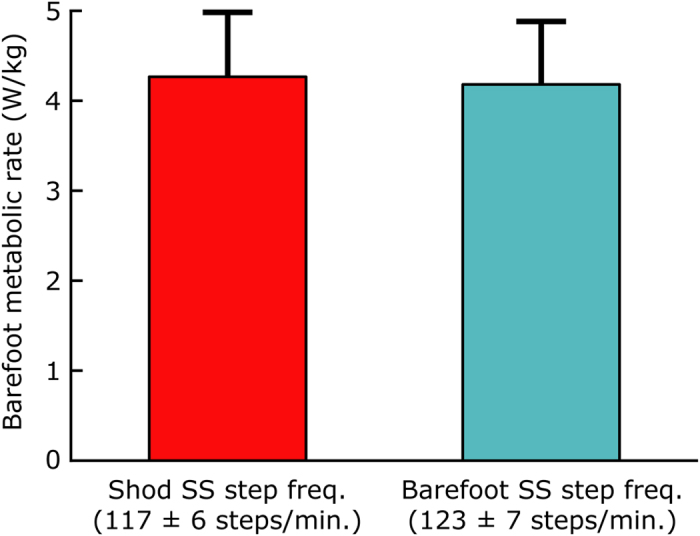
Average metabolic rate of walking barefoot at the shod SS step frequency vs. barefoot SS step frequency. Subjects increased their step frequency when walking barefoot as compared to shod (P = 2e-5). But on average, walking barefoot at the barefoot SS step frequency did not result in reduced metabolic rate compared to barefoot walking at the shod SS frequency (P = 0.40). Inter-subject means and standard deviations are depicted. Paired t-tests were used for statistical comparisons.

We observed that subjects increased their SS step frequency when walking barefoot (122.9 ± 6.5 steps/min, mean ± s.d.) vs. shod (116.5 ± 6.1 steps/min, mean ± s.d.) at 1.4 m/s. The difference (Δ 6.4 ± 2.5 steps/min, mean ± s.d.) was statistically significant (*P* = 2e-5, *N* = 10, Fig. 1). However, on average, we found that the metabolic rate during barefoot walking at the barefoot SS step frequency was not significantly different from the metabolic rate during barefoot walking at the shod SS frequency (*P* = 0.40, *N* = 10, Fig. 1).

**Figure 2:**
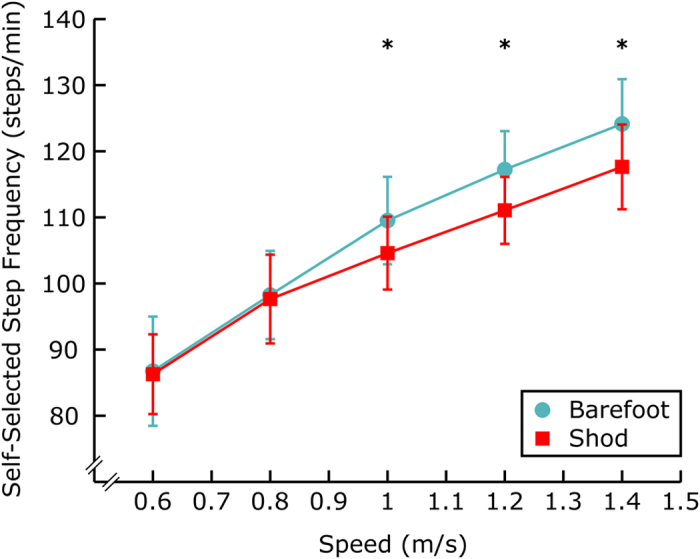
Self-selected step frequency vs. speed. At increasing walking speeds subjects exhibited higher step frequencies barefoot than shod. Barefoot step frequency was significantly higher at speeds at or above 1 m/s (1 m/s, P = 0.002, 1.2 m/s, P = 5e-4, 1.4 m/s, P = 3e-4). Inter-subject means and standard deviations are depicted. Paired t-tests were used for statistical comparisons. Significant differences are denoted with asterisks (^*^).

At speeds of 1 m/s or above, subjects significantly increased their barefoot vs. shod SS step frequency (1 m/s, *P* = 0.002, 1.2 m/s, *P* = 5e-4, 1.4 m/s, *P* = 3e-4, *N* = 8, Fig. 2). However, no significant difference was observed at the slower speeds of 0.6 (*P* = 0.81) and 0.8 m/s (*P* = 0.64, *N* = 8, Fig. 2).

**Figure 3:**
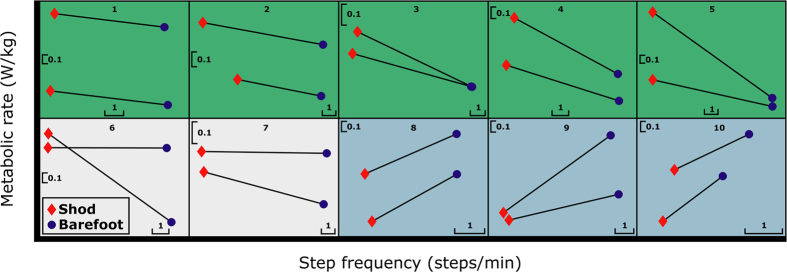
Subject-specific metabolic rate trends for individuals walking barefoot at their shod SS and barefoot SS step frequencies, obtained during an ABBA experimental design. Metabolic trends were observed to be highly subject specific. AB and BA trials are connected with lines to delineate trials that were collected sequentially (i.e., at the beginning or end of the study).

When the metabolic rates for individual subjects were examined, we observed subject-specific trends (Fig. 3). We considered the AB and BA comparisons separately, to avoid bias due to metabolic drift over the course of the experiment. For 5 of the 10 subjects tested, net metabolic cost was consistently lower (−8% average) when they walked barefoot at the barefoot SS step frequency (~123 steps/min) than at the shod SS frequency (~117 steps/min). In contrast, 3 subjects exhibited consistently increased metabolic rate (+10% average) at the barefoot SS frequency. The remaining 2 subjects had an average reduction in metabolic cost (−6%). Technically, both AB and BA trends for these two subjects indicated decreased metabolic cost at barefoot SS frequency. However, in each of these cases, one of the trends was negligible (<0.01 W/kg change in metabolic rate, Fig. 3).

**Figure 4:**
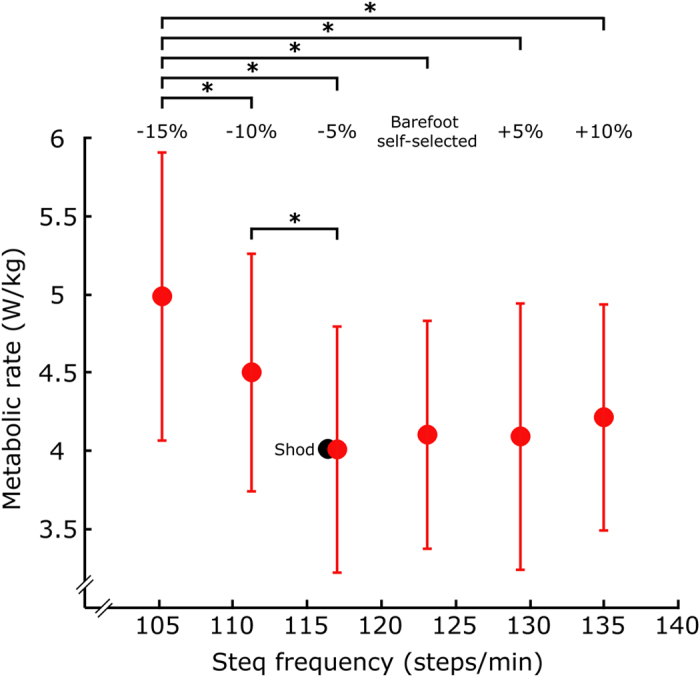
Average metabolic rate for subjects walking barefoot at step frequencies from −15% to +10% relative to their barefoot SS step frequency. The metabolic rate for shod walking at the shod SS frequency is depicted in black. Subject averaged metabolic data resulted in a shallow bowl. Inter-subject means and standard deviations are depicted. Repeated measures analysis of variance (ANOVA) was used for statistical comparisons. Significant differences are denoted with asterisks (^*^).

We found that plotting the average metabolic rate at step frequencies from −15% to +10% relative to SS barefoot frequency resulted in a shallow bowl (U-shaped) profile, qualitatively similar to those reported in previous literature^2^,^4^,^6^. The metabolic rate at the lowest step frequency (−15% relative to barefoot SS) was significantly higher than every other barefoot condition (−10%, *P* = 0.004, −5%, *P* = 2e-7, barefoot SS, *P* = 1e-6, +5%, *P* = 1e-6, +10%, *P* = 1e-5, *N* = 10). The second lowest frequency condition (−10%) was significantly higher than the third lowest (−5%, *P* = 0.003, *N* = 10, Fig. 4). No other statistical differences were observed.

**Figure 5:**
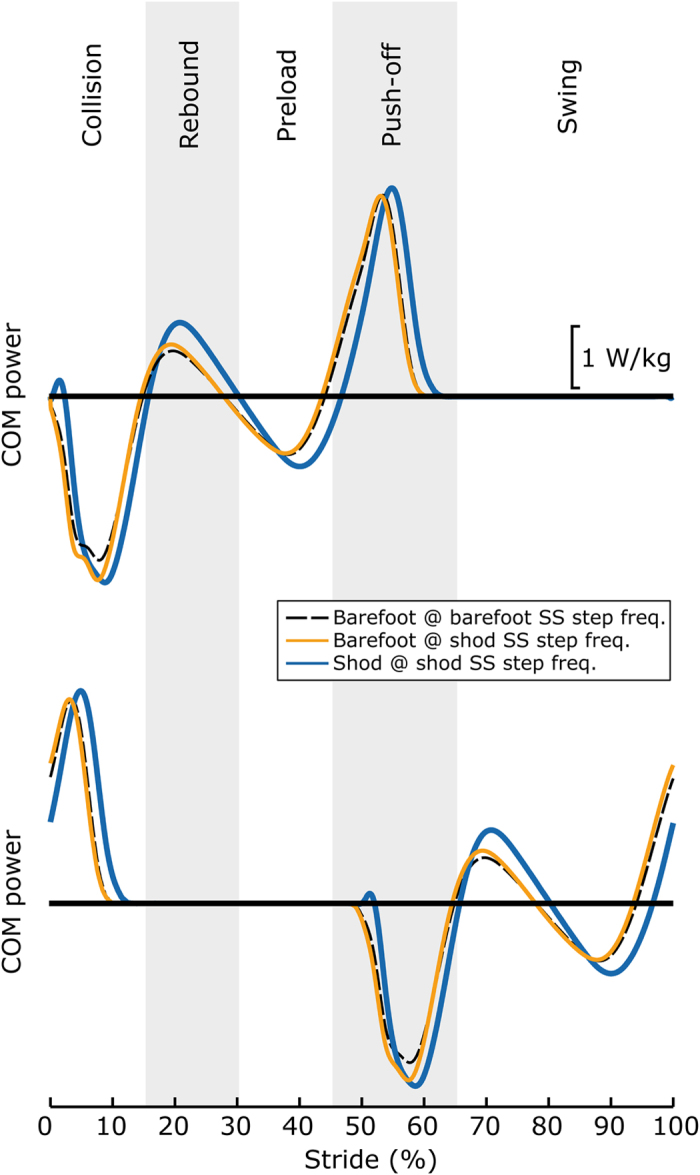
Average center-of-mass (COM) power. Leading (top) and trailing (bottom) limb power curves are depicted separately. Barefoot walking resulted in similar COM power waveforms at both barefoot and shod SS step frequencies when normalized to stride cycle. Shod walking exhibited more substantial differences in COM power, in terms of the magnitude and timing of peaks. The stride cycle is depicted from foot contact to ipsilateral foot contact. Collision, Rebound, Preload, Pushoff and Swing phases of gait are labeled, based on fluctuating regions of positive and negative COM power.

Average COM work rate was similar for barefoot walking at the barefoot and shod SS frequencies when normalized to stride cycle. Changes in mechanical cost of transport were not found to correlate with changes in metabolic cost (R^2^ = 0.004). Larger differences were observed in COM work rate for barefoot walking (at the barefoot and shod SS frequencies) vs. shod walking (at the shod SS frequency, Fig. 5). Barefoot walking exhibited a reduced power transient immediately following heelstrike (~0–2% of stride cycle), compared to shod. The barefoot COM work rate also exhibited reduced peaks during the Rebound and Preload phases of gait^33^,^34^, and the phases of gait were temporally shifted earlier in the stride cycle during barefoot walking, compared to shod (Fig. 5).

## Discussion

Minimization of metabolic energy is often assumed to be the primary factor governing preferred step frequency during human walking and running^10^,^13^,^15^,^16^. This assumption is derived from empirical observations of shod gait, which suggest that people choose a step frequency close to the metabolic minimum during level-ground, forward walking^1^,^2^,^3^,^4^,^5^,^6^ and also during walking conditions when biomechanical demands are altered^11^,^12^. From these observations one might predict that when walking barefoot humans would also adjust their step frequency to minimize metabolic demands. However, our experimental findings did not support this energy minimization hypothesis. All individuals tested increased their step frequency while walking barefoot (by an average of approximately 5% relative to shod, at 1.4 m/s), but on average the metabolic rate for barefoot walking at this barefoot SS frequency was not significantly different than the metabolic rate at the slower shod SS frequency (*P* = 0.40, Fig. 1).

We observed subject-specific trends in our comparison of barefoot walking metabolic rate at the barefoot vs. shod SS frequencies, which could not be directly attributed to changes in mechanical cost of transport. Although five subjects did exhibit a consistent reduction in metabolic rate (−8% average) at their barefoot SS frequency (compared to shod SS frequency), three subjects consistently exhibited the opposite trend (+10% average). For these subjects, their preferred barefoot step frequency during these trials was more metabolically costly at steady state than the step frequency at which they typically walk shod (Fig. 3). We consider these subject-specific metabolic differences potentially meaningful, since they are similar in magnitude to metabolic savings reported in recent exoskeleton studies^35^,^36^ which have been considered human augmentation breakthroughs. The remaining two subjects exhibited less consistent trends; although the AB and BA trends were both technically decreasing, one of them was a negligibly small decrease (Fig. 3). This subset of subjects with less consistent trends may yield additional interesting insights, but the number of individuals observed limited conclusive interpretations from this study.

We have reasonable confidence that the trends observed were not due solely to random variation. Since there are four possible combinations of trends (both up , both down, 1 up/1 down, 1 down/1 up) we would expect due to random chance that 50% of subjects would exhibit consistent trends and 50% would not. However, the probability of all 10 of our subjects having consistent trends by random chance, as we observed, is 0.1% (0.5^10^). Collectively, our results indicate that although subjects consistently increased their step frequency barefoot, the reason for this change in step frequency was not well explained by the minimization of metabolic rate.

We measured metabolic trends twice for each subject (ABBA protocol, Table 1), which was adequate for the purposes of this study, focused on comparing shod vs. barefoot SS step frequencies. To more rigorously quantify the repeatability of these trends a greater number of trials may need to be collected at each step frequency of interest. Alternative experimental designs (e.g., single-subject design^37^,^38^,^39^) may be more appropriate if the primary objective is to assess the consistency of the metabolic trends. Our results add to the scientific knowledge-base of footwear^40^,^41^,^42^ and locomotion studies^35^,^43^,^44^ which indicate highly subject-specific adaptations.

On average, we found no substantial differences in metabolic rate while subjects walked within the range from −5% to +10% of their barefoot SS frequency (Fig. 4); however, the magnitude of this range (15%) is likely an over-estimate for a given individual. Looking only at subject-averaged data suggests that people may be able to choose a relatively wide range of frequencies around the preferred step frequency with minimal or no metabolic penalty. However, because of subject-specific metabolic trends and minima (Fig. 3), averaging across subjects tends to create a shallower metabolic curve on average (Fig. 4). For instance, based solely on the averaged data (Fig. 4) the metabolic difference between shod and barefoot SS frequency is 2%. Yet, if each subject is analyzed separately (Fig. 3) and we consider the absolute value of this metabolic difference, then we find a mean difference of 8%. This corroborates the assertion that subject-specific metabolic bowls are steeper than the average depicted in Fig. 4. Ideally, subject-specific metabolic curves (i.e., metabolic rate vs. step frequency) would be used to determine how much each individual can vary his/her step frequency around the metabolic minimum before incurring a statistically or clinically relevant increase in energy expenditure. But empirically there are practical challenges due to variability in typical metabolic estimates, such as indirect calorimetry^45^. A substantially larger dataset for each individual would be required than was collected in this experiment, and potentially a completely different multi-day experimental design would be necessary (e.g., single-subject design^37^,^38^,^39^). Nevertheless, we can still speculate about the qualitative implications of the metabolic bowl shapes, observed here and in prior studies involving step frequency parameter sweeps^1^,^2^,^3^,^4^,^5^,^6^,^15^,^16^. It is likely that there is some range of step frequencies surrounding the metabolic minimum frequency, at which there is a negligible increase in energy consumption. If so, employing energy minimization as a primary factor in the neuromotor cost function may be beneficial for identifying a subset of economical step frequencies, while other factors or neural constraints might influence the precise step frequency chosen within that range^6^,^13^,^14^,^15^,^16^.

Shoe properties may explain why people adopt a faster step frequency when walking barefoot. For instance, in this study the height of the shoe’s sole added on average 28 mm to the overall length of the leg (height from hip to ground during standing). However, if we apply a published leg length vs. step frequency regression equation^10^ to our data, it only explains a fraction of the change in step frequency (3.1 steps/minute change predicted vs. 6.4 steps/minute change observed). This agrees with previous work that has shown that walking with vs. without shoes can impact the step frequency chosen^7^,^8^,^9^. Other shoe properties such as length, weight, or bending stiffness could also affect preferred barefoot step frequency, and these warrant further study in a controlled experiment.

The absence of shoe cushioning or the perceived level of comfort may also motivate individuals to modify their step frequency during barefoot gait. The “cost of cushioning” hypothesis^18^,^42^,^45^,^46^ posits that muscle contractions help to cushion impacts during locomotion and that there is an energetic penalty incurred for this behavior. Artificial cushioning has been shown to alter foot forces in shod vs. barefoot walking^47^ and when running on compliant vs. stiff track surfaces^23^. Studies of barefoot and shod running (under mass-matched conditions)^18^,^42^,^45^,^46^ and landing barefoot^21^ indicate that in the absence of artificial cushioning (i.e., provided by a shoe or ground surface) individuals tend to expend additional metabolic energy or to perform additional mechanical work, presumably to avoid discomfort associated with the higher impacts experienced by the musculoskeletal system in barefoot activities. This observed phenomenon may also be relevant to footstrikes experienced during barefoot walking, and we observed some indirect biomechanical evidence that footstrike cushioning and/or subjective comfort may indeed be key factors underlying the barefoot SS step frequency. For instance, we observed that the SS step frequency for barefoot walking was only considerably faster than shod walking at or above 1 m/s (Fig. 2). This indicates that as speed increases during barefoot gait that people elect to take shorter steps than they would shod, potentially to reduce footstrike collision magnitudes. However, there were a variety of other differences observed in the COM work rate between shod and barefoot walking (Fig. 5), indicating that humans also make other biomechanical adjustments. Although shoe properties and subjective comfort may be critical factors underlying subject-specific behavioral modifications during locomotion, the challenge remains to integrate these concepts into biomechanical models and our theoretical understanding of gait, and to account for these factors when designing future experimental studies.

These findings have important translational, methodological and scientific implications. Translationally, these results indicate that for some individuals it may be possible to improve their barefoot gait economy through biofeedback or training. It has previously been demonstrated that individuals could be given feedback to reduce their metabolic cost while walking down a slope^48^. The same may be true for barefoot walking and potentially running. If some individuals are operating close to but not at their metabolic minimum step frequency, then it is plausible that footwear properties could be manipulated to encourage a more optimal step frequency, which would be expected to reduce metabolic demands and improve an individual’s performance^5^. Methodologically, this study highlights the subject-specific nature of locomotor adaptations, even during relatively small deviations from shod walking. Averaging results across subjects may obscure certain subject-specific trends and may not reflect individual gait adaptations, which can affect scientific interpretations. Similar subject-specific results have been observed in previous footwear^40^,^41^,^42^ and assistive device studies^35^,^43^,^44^. This presents a nuanced challenge for data analysis and interpretation, and calls attention to limitations of commonly-used repeated measures study designs. Alternative experimental designs, such as single-subject design^37^,^38^,^39^, may be beneficial for discerning how individuals respond to different interventions. These approaches could also be highly relevant to clinical populations, given inter-individual variability.

This study has several limitations. First, all subjects tested were habitually shod walkers. Second, subjects wore their daily tennis shoes, but we did not standardize on a single brand/type of shoe worn in the study. Nevertheless, all subjects demonstrated the same increasing trend in step frequency when they transitioned to barefoot walking, so this footwear confound is not expected to change any of our qualitative findings. Third, this study focused on metabolic rate as the primary outcome measure, which can exhibit variability due to physiological and environmental factors^45^. For the main comparison of interest (Figs 1 and 3), this was partly controlled for by using an ABBA experimental design to identify consistent trends. However, for other conditions metabolic data were only collected in one trial. Additionally, metabolic data were only collected for one walking speed, limiting our ability to fully generalize conclusions. Ground reaction force data were collected and analyzed for this study, but not kinematics or electromyography, limiting identification of specific mechanisms underlying step frequency changes. Prior barefoot studies have also found other differences in shod vs. barefoot gait, which were not re-investigated in our analysis, including differences in pressure patterns^49^ and foot motion^9^,^50^.

It is commonly stated in literature that humans naturally select a step frequency that minimizes metabolic cost while walking; however, upon reviewing the primary sources we found that this statement is based on somewhat limited data and is not convincingly validated. We identified several primary sources, which empirically varied step frequency and measured metabolic cost during shod walking, and which have been frequently cited in support of the assertion that humans select a step frequency that minimizes metabolic cost during walking. Three often-cited studies are Atzler^4^ (*N* = 1, data reproduced in Elftman^3^), Cotes^16^ (*N* = 1), and Molen^6^ (*N* = 1 for comprehensive parameter sweep, *N* = 10 for reduced parameter sweep, data reproduced in Zarrugh^1^). However, these studies did not rigorously compare metabolic cost at SS step frequency vs. alternative step frequencies. Also, in each of these studies a relatively small number of frequencies were tested over a large range. For instance, Atzler tested step frequencies of 50, 75, 100, 130 and 150 steps/minute. In contrast, our study only encompassed frequencies around SS, ranging from ~105–135 steps/minute (Fig. 4, depicting frequencies from −15% to +10% of relative to barefoot SS step frequency). Thus, in Atlzer, Cotes, and Molen, limited data were collected around the metabolic minimum step frequency, yielding insufficient data to validate the claim that individuals adopt a step frequency that minimizes metabolic cost. A fourth study by Zarrugh^2^ performed a multi-subject experiment (*N* = 7) that concluded “the freely chosen step rate requires the least oxygen consumption at any given speed.” However, upon careful inspection of the data we observe that for several subjects their SS step frequency did not correspond with the minimum measured metabolic cost. Despite the study’s stated conclusion, the data did not definitively demonstrate that SS step frequency resulted in a lower metabolic rate than other faster or slower step frequencies. Rather results and analyses were focused on validating a previous predictive walking model^1^. A fifth study by Holt^5^ tested 8 subjects walking with a range of step frequencies (up to ±15 steps/minute from SS) at constant speed. They observed that a quadratic trend (i.e., bowl shape) could be fit to the mean metabolic curve (individual subject results were not reported). Although the authors posited that the SS step frequency resulted in minimum metabolic cost, the results reported did not actually indicate that the metabolic cost at preferred step frequency was statistically lower than step frequencies that were slightly faster or slower (e.g., ±5 steps/minute from SS). Results from our study indicate that changes in step frequency on the order of 5 steps/minute could have a meaningful effect on metabolic energy consumption (Fig. 3). It therefore remains unclear if people prefer the metabolically optimal step frequency, or simply a frequency near the metabolic minimum. We note that metabolic effects could be indirect, and that the changes in metabolic cost observed could be the result of an optimization of many task dependent factors including step frequency. In a follow-up study by Holt^51^, a larger range of frequencies were tested, but these were relatively far from the SS frequency (±15%, 25% and 35% of SS). A study by Minetti^52^ also measured metabolic rate at SS and other controlled step frequencies (*N* = 6). However, it only reported cubic regressions fit to the mean metabolic data (R-squared fit values ranging from 0.391 to 0.774), and based on the published data it is not possible to determine if the SS frequency is at, or simply near the metabolic minimum for each subject tested. The most comprehensive study by Bertram^15^ tested 10 individuals at 49 combinations of speed (0.26–2.34 m/s) and step frequency (~48–176 steps/min). The study found a reasonable correspondence between the SS step frequency and the metabolically optimal frequency; however, it ultimately concluded that notable deviations from the metabolic minimum suggested that other factors also influence the SS gait pattern. Each of the studies summarized above is consistent with the notion that people generally prefer a step frequency relatively near to the metabolic minimum frequency; however, direct empirical evidence is lacking to either support or refute the claim that humans naturally adopt a step frequency that actually minimizes metabolic cost. Additional studies have considered the effect of step frequency on mechanical energy outcomes^13^, or used modeling approaches to consider the energy minimization hypothesis^10^,^14^, but these studies did not directly quantify metabolic cost in human subjects. Results from our current study (which we remind was on barefoot and not shod walking) suggest subject-specific metabolic trends in which the SS step frequency is not always energetically optimal. Further experimental studies, which perform multiple repeated tests on a subset of conditions to identify consistent relative differences in metabolic rate, may be necessary to conclusively address whether individuals actually prefer the optimal (metabolic minimum) step frequency, or to identify the degree to which individuals deviate from this metabolically optimal gait pattern.

In summary, we found that at speeds at or above 1 m/s individuals consistently increased their step frequency when walking barefoot, as compared to shod. However, contrary to the energy minimization hypothesis, the observed increase in barefoot step frequency was not well explained by a ubiquitous desire to minimize metabolic cost. While walking barefoot, half of our subjects demonstrated a consistent reduction in metabolic rate at the barefoot SS frequency as compared to the shod SS frequency. However, about a third of the subjects consistently exhibited the opposite trend – their preferred barefoot step frequency was actually more metabolically costly than their typical shod step frequency. For these individuals it may be possible to use biofeedback to improve their barefoot walking economy. Biomechanical observations suggest that shoe properties and/or perceived comfort may be important factors influencing preferred barefoot step frequency, and these warrant further study through controlled experiments. Overall, these findings indicate subject-specific behavioral adaptations during barefoot gait, which are not well captured by group-averaged data. Based on the observations in this study it does not appear that all individuals optimize their step frequency to minimize metabolic cost during barefoot walking. Furthermore, based on a re-evaluation of prior literature, the degree to which shod walkers minimize their metabolic cost also remains unclear.

## Additional Information

**How to cite this article**: Yandell, M. B. and Zelik, K. E. Preferred Barefoot Step Frequency is Influenced by Factors Beyond Minimizing Metabolic Rate. *Sci. Rep.* **6**, 23243; doi: 10.1038/srep23243 (2016).

## Figures and Tables

**Table 1:** Experimental Protocol.

Pre-Test	0	1	2	3–8	9	10	Post-Test
Treadmill Acclimation, Measurement of SS Step Freq. Barefoot & Shod	Standing at Rest	A	B	Barefoot walking at freqs. −15%, −10%, −5%, +5% & +10% from Barefoot SS freq., & Shod walking at Shod SS freq.	B	A	Re-measure SS. Step Freq. Barefoot & Shod

A: Barefoot walking at Barefoot SS Step Frequency, and B: Barefoot walking at Shod SS Step Frequency.
